# Novel haemodialysis (HD) treatment employing molecular hydrogen (H_2_)-enriched dialysis solution improves prognosis of chronic dialysis patients: A prospective observational study

**DOI:** 10.1038/s41598-017-18537-x

**Published:** 2018-01-10

**Authors:** Masaaki Nakayama, Noritomo Itami, Hodaka Suzuki, Hiromi Hamada, Ryo Yamamoto, Kazumasa Tsunoda, Naoyuki Osaka, Hirofumi Nakano, Yukio Maruyama, Shigeru Kabayama, Ryoichi Nakazawa, Mariko Miyazaki, Sadayoshi Ito

**Affiliations:** 1 Tohoku University, United Centers for Advanced Research and Translational Medicine, Center for Advanced and Integrated Renal Science, Sendai, Japan; 2 Tohoku University Hospital, Research Division of Chronic Kidney Disease and Dialysis Treatment, Sendai, Japan; 3 Fukushima Medical University, Department of Nephrology and Hypertension, Fukushima, Japan; 4 Nikko-Memorial Hospital, Kidney Center, and Higashi Muroran Satellite Clinic, Muroran, Japan; 5 Horai-Higashi Clinic Fukushima, Fukushima, Japan; 6 Tateishi-Jin Clinic, Tokyo, Japan; 7 Noboribetsu Memorial Hospital, Noboribetsu, Japan; 8 Gumyoji-Jin Clinic, Yokohama, Japan; 9 Kashima Hospital, Dialysis Center, Iwaki, Japan; 10 The Tokyo Jikei University School of Medicine, Department of Nephrology and Hypretension, Tokyo, Japan; 11 Tokatsu Clinic Mirai, Matsudo, Japan

## Abstract

Recent studies have revealed unique biological characteristics of molecular hydrogen (H_2_) as an anti-inflammatory agent. We developed a novel haemodialysis (E-HD) system delivering an H_2_ (30–80 ppb)-enriched dialysis solution by water electrolysis, and conducted a non-randomized, non-blinded, prospective observational study exploring its clinical impact. Prevalent chronic HD patients were allocated to either the E-HD (n = 161) group or the conventional HD (C-HD: n = 148) group, and received the respective HD treatments during the study. The primary endpoint was a composite of all-cause mortality and development of non-lethal cardio-cerebrovascular events (cardiac disease, apoplexy, and leg amputation due to peripheral artery disease). During the 3.28-year mean observation period, there were no differences in dialysis parameters between the two groups; however, post-dialysis hypertension was ameliorated with significant reductions in antihypertensive agents in the E-HD patients. There were 91 events (50 in the C-HD group and 41 in the E-HD group). Multivariate analysis of the Cox proportional hazards model revealed E-HD as an independent significant factor for the primary endpoint (hazard ratio 0.59; [95% confidence interval: 0.38–0.92]) after adjusting for confounding factors (age, cardiovascular disease history, serum albumin, and C-reactive protein). HD applying an H_2_-dissolved HD solution could improve the prognosis of chronic HD patients.

## Introduction

The combination of enhanced oxidative stress and inflammation in patients on chronic haemodialysis (HD) treatment plays a crucial role in the occurrence of excessive cardiovascular events and death^1,2^. The bio-incompatibility of the HD procedure is supposed to be involved with this pathology. HD may exaggerate leukocyte activation and injury^3–5^, which enhance oxidative stress and inflammation. Therefore, we hypothesized that ameliorating the stress to leukocytes during HD may have a beneficial effect on patient outcomes.

Molecular hydrogen (H_2_) is an inert gas with no known side effects. Recent studies have shown that H_2_ acts as an antioxidant and an anti-inflammatory agent, and ameliorates cellular and organ damage^6,7^. We therefore developed a novel HD system using highly dissolved H_2_ water rendered by the water electrolysis technique^8–10^. Previous pilot studies, including ours, have reported that suppression of interleukin-6, high-sensitivity C-reactive protein (CRP), monocyte chemoattractant protein-1 (MCP-1)/chemokine (C-C motif) ligand 2 (CCL2), and myeloperoxidase (MPO), decrease oxidative injury of lymphocytes, improve the redox status of serum albumin, and ameliorate hypertension^8–14^. In reference to these findings, we conducted a non-randomized, non-blinded, prospective observational study to compare the outcomes between patients receiving haemodialysis using an H_2_-enriched dialysis solution (E-HD group) and patients receiving conventional haemodialysis (C-HD group).

## Results

### Patient registration and characteristics

**Figure 1:**
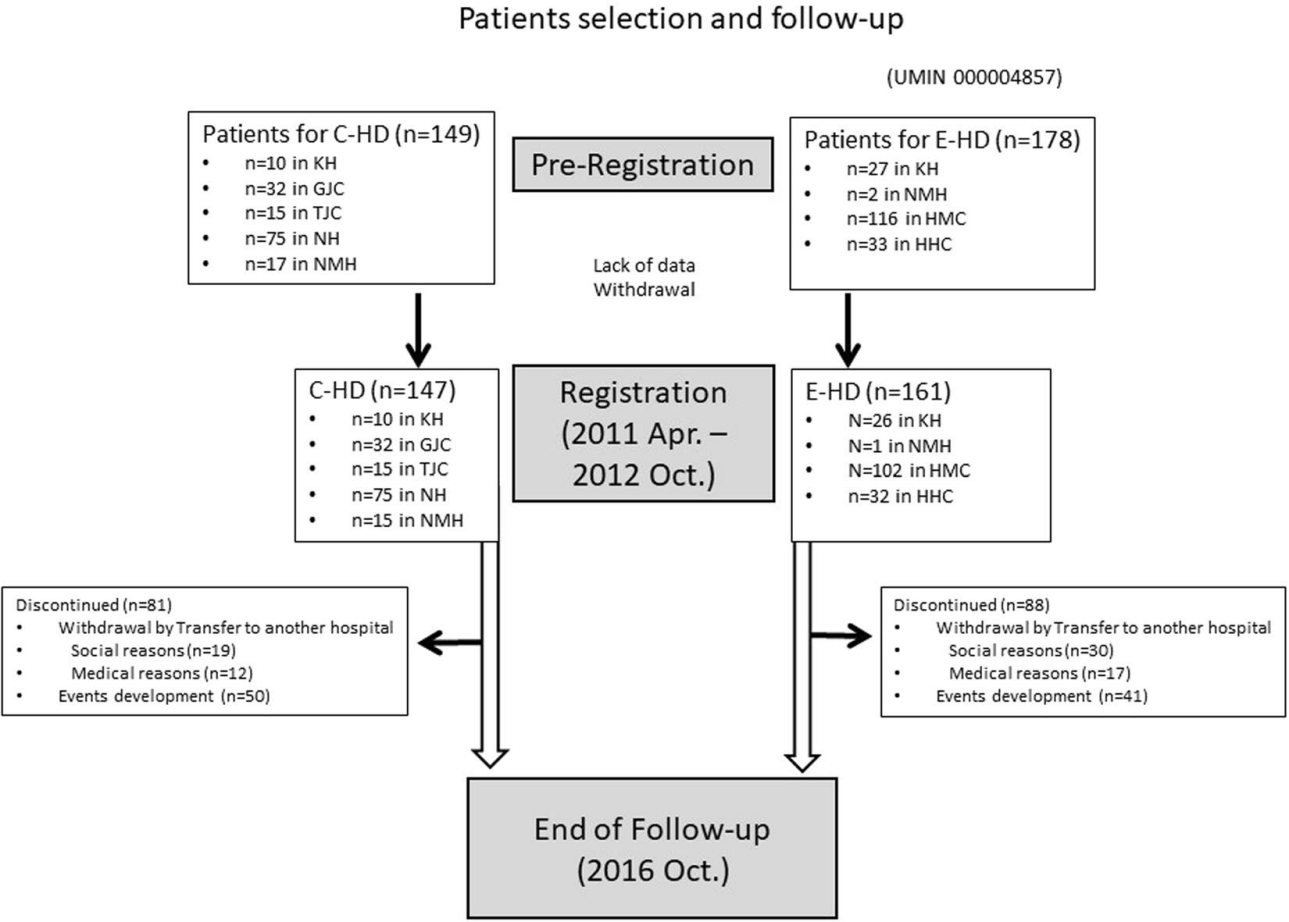
Flow chart from pre-registration to the end of observation. Abbreviations: C-HD, conventional haemodialysis; E-HD, electrolyzed water haemodialysis; KH, Kashima Hospital; GJC, Gumyoji Jin Clinic; TJC, Tateishi Jin Clinic; NH, Noboribetsu Hospital; NMH, Nikko Memorial Hospital; HMC, Higashi Muroran Clinic; HHC, Higashi Horai Clinic.

Patients were recruited during April 2011 and October 2012. Of the 327 prevalent chronic HD patients who were pre-registered, 18 were excluded because of the lack of data and withdrawal. Ultimately, 148 patients were allocated to the C-HD group and 161 patients were allocated to the E-HD group (Fig. 1). The patients’ characteristics in the two groups at baseline are shown in Table 1. All subjects were treated by the standard HD schedule (three sessions/week, 4–5 h/session), using high-performance biocompatible dialyzers with fixed blood flow rate (QB) (200 mL/min) and dialysate flow rate (QD) (500 mL/min). Patients who had been treated by a vitamin-E coated dialyzer were excluded from this study. At baseline, there was no statistical difference between the groups in the blood urea nitrogen (BUN) reduction rate by HD (69.7 ± 6.9% in the C-HD group and 70.3 ± 8.4% in the E-HD group; p = 0.485).

**Table 1:** Patient characteristics.

Characteristic	C-HD	E-HD	*P* Value
*N*	148	161	—
Age (y)	67.4 ± 11.8	64.0 ± 11.9	<0.05
Gender, male (%)	92 (62.2)	85 (52.8)	NS
Dialysis vintage (months)	60 (3, 263)	80 (2, 478)	<0.01
Cause of renal failure (DM, (%))	62 (41.9)	55 (34.2)	NS
Patients with CVD history (%))	36 (24.3)	53 (32.9)	NS
with multiple CVDs (%)	5 (3.4)	10 (6.2)	NS
with cardiac disease (%)	25 (16.9)	31 (19.3)	NS
with apoplexy (%)	11 (7.4)	29 (18.0)	<0.01
with PAD (%)	5 (3.4%)	3 (1.9%)	NS
Body weight (pre HD, kg)	59.3 ± 12.0	58.9 ± 11.2	NS
Body weight (post HD, kg)	57.0 ± 11.7	56.3 ± 10.9	NS
CTR (%)	48.7 ± 6.0	48.7 ± 5.5	NS
Pre-dialysis SBP (mmHg)	154 ± 27	154 ± 25	NS
Pre-dialysis DBP (mmHg)	79 ± 15	80 ± 16	NS
Post-dialysis SBP (mmHg)	142 ± 24	135 ± 24	<0.05
Post-dialysis DBP (mmHg)	75 ± 14	73 ± 14	NS
Patients on Anti-hypertensive agents (%)	108 (73.0)	107 (66.5)	NS
Patients with ESA (%)	124 (83.8)	140 (87.0)	NS
Fatigue Grade	2.9 ± 1.0	2.9 ± 1.1	NS
Pruritis Intensity Grade	3.4 ± 0.9	3.2 ± 0.9	<0.05
Puriritis Frequency Grade	3.2 ± 1.0	3.0 ± 1.1	NS

C-HD, conventional haemodialysis; E-HD, electrolyzed water haemodialysis.

CVD, cardio-cerebrovascular disease; HD, haemodialysis; PAD, peripheral arterial disease; SBP, systolic blood pressure; DBP, diastolic blood pressure; ESA, erythropoiesis stimulating agents.

### Changes in laboratory and subjective/objective parameters during the study

**Figure 2:**
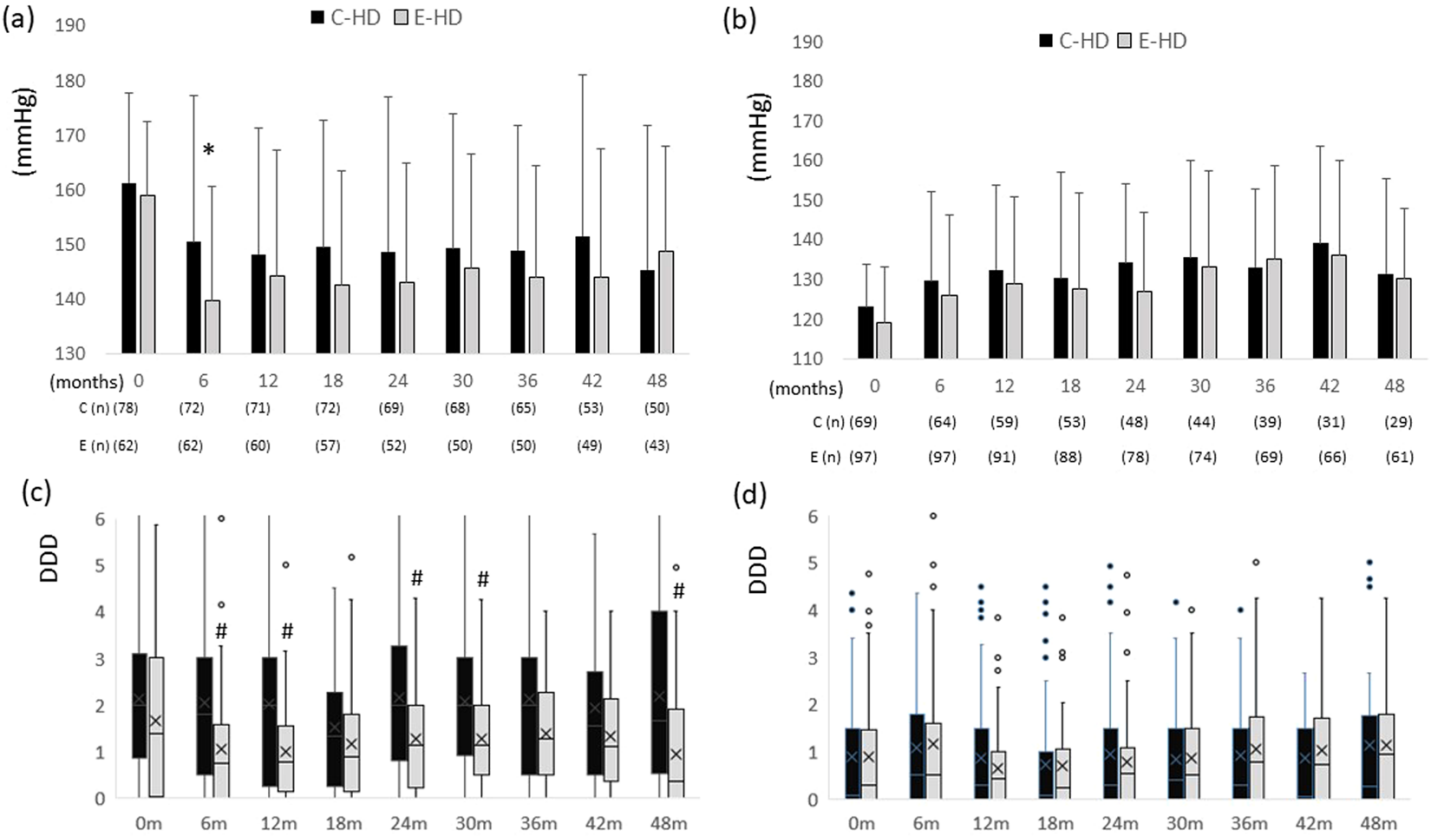
Changes in post-dialysis systolic blood pressure, and prescription of antihypertensive agents during the study. Patients with post-dialysis SBP ≥ 140 mmHg (n = 139) at baseline (0 month): changes in post-dialysis SBP (**a**), and changes in DDD (**b**); Patients with post-dialysis SBP < 140 mmHg (n = 168) at baseline: changes in post-dialysis SBP (**c**), and changes in DDD (**d**). Abbreviations: C-HD, conventional haemodialysis; E-HD, electrolyzed water haemodialysis; SBP, systolic blood pressure; DDD, daily defined dose of antihypertensive agents. (**a**,**c**) There were significant differences in post-dialysis SBP (6 months; p < 0.05), and DDD (6, 12, 18 months; p < 0.05, respectively) between the two groups. (**b**,**d**) No differences were observed in post-dialysis SBP or DDD between the two groups.

HD-related laboratory parameters at the time of the first HD session of the respective weeks are shown in Table 2. No differences were noted between the two groups during the study period. Regarding subjective symptoms, there was a significant difference in the grade of pruritus between the two groups at baseline (with more severe symptoms in the E-HD group); however, no differences were found during the course of the study. Small but significant differences were noted between the two groups in the fatigue grade (fewer symptoms in the E-HD group) at 48 weeks. No differences were observed in the time-course pre-dialysis blood pressures (BPs); however, post-dialysis BPs differed between the two groups. In sub-analysis of the post-dialysis systolic BP (SBP) levels at baseline, there were significant differences in post-dialysis SBP (6 months) and Defined Daily Dose^15^ of antihypertensive medication (6, 12, 18 months) in patients with post-dialysis SBP ≥ 140 mmH at baseline, while no statistical differences were found in those parameters in patients with post-dialysis SBP < 140 mmHg (Fig. 2).

**Table 2:** Dialysis-related and subjective/objective parameters in the two groups.

Months		0 m	6 m	12 m	18 m	24 m	30 m	36 m	42 m	48 m
WBC count (/µL)	C-HD	5504 ± 1653	5597 ± 1840	5461 ± 1669	5321 ± 1778	5251 ± 1996	5404 ± 2093	5701 ± 2014	5543 ± 1840	5541 ± 1985
	(n)	148	136	128	126	117	109	104	84	80
	E-HD	5852 ± 1803	5865 ± 2091	5734 ± 2083	5648 ± 1851	5779 ± 1823	5584 ± 1751	5620 ± 1684	5637 ± 1759	5642 ± 1793
	(n)	161	160	152	145	131	123	121	112	105
Hemoglobin (g/dL)	C-HD	10.6 ± 1.1	10.6 ± 1.2	10.4 ± 1.3	10.7 ± 1.4	10.4 ± 1.3	10.5 ± 1.3	10.4 ± 1.3	10.6 ± 1.3	10.7 ± 1.3
	(n)	148	136	128	126	117	109	104	83	80
	E-HD	11.1 ± 1.2	11.0 ± 1.0	10.7 ± 1.2	10.9 ± 1.2	10.4 ± 1.3	11.1 ± 1.1	10.8 ± 1.1	10.9 ± 1.1	11.1 ± 1.3
	(n)	161	159	152	145	131	123	121	112	105
BUN (mg/dL)	C-HD	66.8 ± 15.1	63.7 ± 15.0	65.3 ± 13.9	56.1 ± 14.5	58.8 ± 14.3	56.3 ± 14.0	61.3 ± 13.1	57.0 ± 14.0	61.1 ± 13.7
	(n)	148	136	128	126	117	109	103	84	80
	E-HD	69.0 ± 15.8	67.5 ± 16.5	65.2 ± 15.5	62.9 ± 15.8	64.3 ± 14.5	61.0 ± 13.2	62.5 ± 15.1	63.0 ± 14.8	61.4 ± 13.4
	(n)	161	160	152	145	131	123	121	112	105
creatinine (mg/dL)	C-HD	10.8 ± 2.6	11.1 ± 2.5	10.9 ± 2.5	10.0 ± 2.3	10.3 ± 2.3	10.4 ± 2.5	10.9 ± 2.5	11.0 ± 2.4	10.8 ± 2.5
	(n)	148	136	128	126	117	110	104	84	80
	E-HD	10.6 ± 3.0	10.4 ± 2.8	10.7 ± 2.8	10.3 ± 2.8	10.6 ± 2.6	10.7 ± 2.6	10.4 ± 2.3	10.8 ± 2.2	10.6 ± 2.4
	(n)	161	159	152	145	131	123	121	112	105
Ca (mg/dL)	C-HD	8.8 ± 0.7	8.8 ± 0.8	8.8 ± 0.8	8.8 ± 0.6	8.8 ± 0.7	8.8 ± 0.7	8.8 ± 0.7	8.9 ± 0.8	8.6 ± 0.8
	(n)	148	136	128	126	117	110	104	84	79
	E-HD	8.8 ± 0.7	8.8 ± 0.6	8.7 ± 0.7	8.8 ± 0.6	8.7 ± 0.7	8.8 ± 0.6	8.8 ± 0.7	8.8 ± 0.6	8.8 ± 0.6
	(n)	160	159	152	145	131	123	121	112	105
Pi (mg/dL)	C-HD	5.5 ± 1.3	5.5 ± 1.4	5.6 ± 1.4	5.5 ± 1.3	5.6 ± 1.3	5.3 ± 1.3	5.7 ± 1.4	5.5 ± 1.6	5.8 ± 1.4
	(n)	148	136	128	126	117	109	104	84	80
	E-HD	5.6 ± 1.4	5.6 ± 1.5	5.4 ± 1.3	5.4 ± 1.3	5.4 ± 1.4	5.4 ± 1.1	5.4 ± 1.1	5.3 ± 1.3	5.2 ± 1.1
	(n)	161	161	154	147	133	125	123	114	107
B2-microglobulin (mg/L)	C-HD	27.7 ± 7.0	28.2 ± 6.6	27.5 ± 6.4	26.9 ± 5.8	26.6 ± 6.0	27.5 ± 5.3	29.9 ± 5.8	29.8 ± 5.7	29.1 ± 6.0
	(n)	148	131	126	126	116	108	102	80	78
	E-HD	26.9 ± 6.5	27.0 ± 6.9	27.6 ± 6.5	26.0 ± 5.9	26.9 ± 6.3	27.3 ± 5.6	28.4 ± 5.6	28.2 ± 5.7	28.6 ± 5.3
	(n)	161	159	149	142	131	122	120	110	104
CRP (mg/dL)	C-HD	0.32 ± 0.57	0.23 ± 0.34	0.41 ± 0.93	0.53 ± 2.24	0.26 ± 0.44	0.40 ± 0.95	0.45 ± 0.97	0.99 ± 5.12	0.82 ± 2.10
	(n)	148	133	128	126	115	109	101	81	78
	E-HD	0.39 ± 0.73	0.45 ± 1.03	0.66 ± 1.52	0.56 ± 1.87	0.57 ± 1.17	0.38 ± 0.88	0.41 ± 0.71	0.35 ± 0.67	0.62 ± 1.91
	(n)	161	160	152	145	131	123	121	112	105
albumin (g/dL)	C-HD	3.5 ± 0.3	3.6 ± 0.3	3.6 ± 0.4	3.5 ± 0.3	3.5 ± 0.3	3.5 ± 0.4	3.5 ± 0.3	3.5 ± 0.3	3.4 ± 0.3
	(n)	148	136	126	124	116	109	103	83	79
	E-HD	3.7 ± 0.3	3.6 ± 0.3	3.7 ± 0.4	3.5 ± 0.4	3.5 ± 0.3	3.6 ± 0.3	3.5 ± 0.3	3.6 ± 0.3	3.6 ± 0.3
	(n)	161	159	152	145	131	123	121	112	107
Dry weight (kg)	C-HD	56.6 ± 11.8	57.0 ± 11.6	57.6 ± 12.3	57.0 ± 11.6	56.9 ± 11.4	56.8 ± 11.1	56.6 ± 11.5	56.4 ± 12.6	56.4 ± 12.3
		147	140	133	129	119	114	106	87	82
	E-HD	56.4 ± 10.9	56.5 ± 11.0	56.5 ± 11.4	56.3 ± 11.5	56.9 ± 11.8	56.4 ± 11.3	56.5 ± 11.3	56.5 ± 11.6	58.3 ± 12.2
	(n)	161	160	152	146	131	125	120	113	107
CTR (%)	C-HD	48.7 ± 6.0	49.1 ± 4.2	49.0 ± 4.2	49.0 ± 4.4	49.9 ± 5.3	49.6 ± 5.2	49.7 ± 5.2	49.5 ± 5.8	49.1 ± 6.2
	(n)	148	134	131	115	117	112	104	84	79
	E-HD	48.7 ± 5.5	49.0 ± 5.4	49.3 ± 5.6	49.4 ± 5.4	49.2 ± 5.3	49.3 ± 5.4	49.5 ± 5.6	48.7 ± 5.4	49.0 ± 5.1
	(n)	161	155	148	133	129	123	119	108	101
pre-dialysis MBP (mmHg)	C-HD	104 ± 17	97 ± 16	104 ± 15	100 ± 14	100 ± 16	101 ± 17	104 ± 15	101 ± 18	101 ± 18
	(n)	148	137	121	112	101	88	78	66	62
	E-HD	103 ± 22	94 ± 19	103 ± 18	102 ± 19	103 ± 19	105 ± 15*	105 ± 15	104 ± 16	106 ± 18
	(n)	161	163	152	146	131	125	120	115	105
post-dialysis MBP (mmHg)	C-HD	97 ± 13	93 ± 18	96 ± 13	96 ± 15	96 ± 13	98 ± 14	98 ± 12	100 ± 12	95 ± 12
	(n)	148	137	121	112	101	88	78	66	62
	E-HD	93 ± 20	90 ± 18	94 ± 16	92 ± 16*	92 ± 15**	95 ± 16	95 ± 14*	96 ± 16	95 ± 13
	(n)	161	162	152	146	131	125	120	115	105
DDD	C-HD	1.04	1.03	1.00	1.00	1.22	1.36	1.34	1.12	1.00
		(0, 2.34)	(0, 2.53)	(0, 2.05)	(0, 2.00)	(0, 2.83)	(0.18, 2.33)	(0, 2.50)	(0, 2.05)	(0.02, 2.71)
	(n)	147	137	130	127	118	112	105	86	84
	E-HD	0.57	0.57*	0.5**	0.50	0.76**	0.81*	1.07	0.86	0.62*
		(0, 2.14)	(0, 1.53)	(0, 1.21)	(0, 1.34)	(0, 1.50)	(0.03, 1.62)	(0.06, 1.90)	(0, 1.87)	(0, 1.62)
	(n)	159	159	151	145	130	124	120	115	104
Fatigue Grade	C-HD	2.9 ± 1.0	2.8 ± 1.1	2.6 ± 1.1	3.0 ± 1.2	2.8 ± 1.2	2.7 ± 1.2	2.8 ± 1.2	2.9 ± 1.1	2.9 ± 1.1
	(n)	148	136	124	123	111	112	103	79	74
	E-HD	2.9 ± 1.1	3.0 ± 1.0	2.9 ± 1.2	2.9 ± 1.3	2.9 ± 1.3	3.1 ± 1.1*	2.9 ± 1.4	3.0 ± 1.3	3.2 ± 1.1
	(n)	161	152	139	136	124	120	118	106	96
Pruritus Intensity Grade	C-HD	3.4 ± 0.9	3.2 ± 0.9	3.1 ± 1.0	3.2 ± 1.0	3.1 ± 1.1	3.1 ± 1.0	3.1 ± 1.0	3.2 ± 0.9	3.0 ± 1.0
	(n)	148	136	124	123	110	112	103	79	74
	E-HD	3.2 ± 0.9*	3.2 ± 1.1	3.4 ± 0.9	3.5 ± 0.9	3.2 ± 1.0	3.4 ± 0.9	3.3 ± 1.0*	3.4 ± 0.9	3.3 ± 0.9*
	(n)	161	152	139	136	124	120	118	106	96
Puriritus Frequency Grade	C-HD	3.2 ± 1.0	2.9 ± 1.1	2.9 ± 1.1	2.9 ± 1.2	2.9 ± 1.2	2.9 ± 1.2	2.9 ± 1.1	3.1 ± 1.1	2.8 ± 1.2
	(n)	148	135	124	123	111	112	103	79	74
	E-HD	3.0 ± 1.1	3.1 ± 1.2	3.2 ± 1.1	3.3 ± 1.0	3.1 ± 1.1	3.3 ± 1.0	3.2 ± 1.1	3.3 ± 1.1	3.2 ± 1.1*
	(n)	161	152	139	136	124	120	118	106	96

vs. C-HD; *p < 0.05, **p < 0.01

MBP, mean blood pressure; CTR, cardiothoracic ratio; DDD, defined daily dose of anti-hypertensive agents.

C-HD, conventional haemodialysis; E-HD, electrolyzed water haemodialysis; WBC, white blood cell; BUN, blood urea nitrogen; Ca, serum Calcium; Pi, serum phosphate; CRP, C-reactive protein.

### Composite events summary and multivariate analysis of risk factors for events

**Figure 3:**
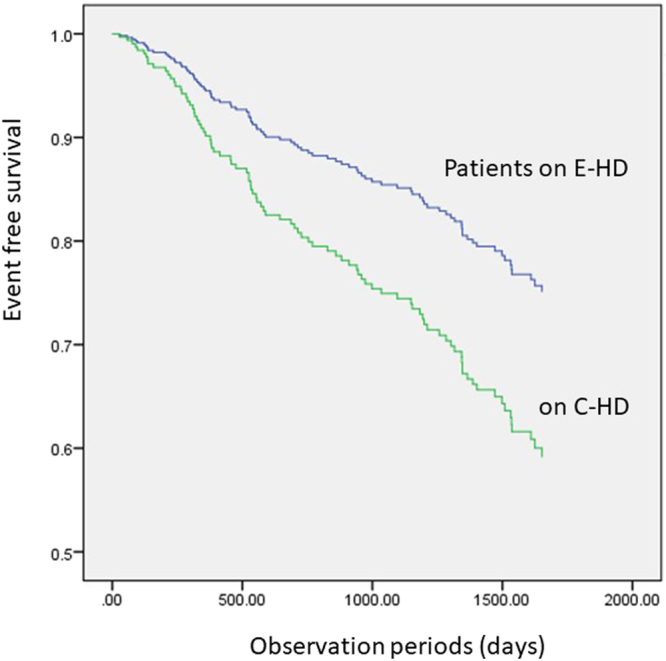
Cox proportional hazards model demonstrating events-free differences between patients on C-HD and those on E-HD. Treatment with E-HD was an independent predicting factor for events (hazard ratio:0.593; p < 0.05). Abbreviations: C-HD, conventional haemodialysis; E-HD, electrolyzed water haemodialysis.

During the mean observation period of 3.28 years, there were 91 events: 50 in the C-HD group and 41 in the E-HD group (Table 3). In Cox proportional hazards model analysis, possible risk factors for the primary endpoints, which were identified via p-values < 0.1, were depicted, e.g., E-HD dialysis modality, age, history of cardio-cerebrovascular disease (CVD), serum albumin, and CRP. Multivariate analysis after adjusting for these factors revealed E-HD as an independent significant factor for the primary event (hazard ratio [HR] 0.59 [95% confidence interval [CI]: 0.38–0.92]) (Fig. 3 and Table 4).

**Table 3:** Summary of events in the two groups.

	C-HD	E-HD
Observation vintage (patient⋅year)	467	544
Number of Primary events	50	41
(all causes of deaths and non-lethal CVD events)		
Cardiac events including death	29	20
Congestive heart failure	11	8
Ischemic heart disease	13	9
Aortic aneurysm rupture	1	1
Sudden cardiac arrest	4	2
Apoplexy including death (bleeding/infarction)	6 (1/5)	10 (2/8)
PAD including death	8	2
Primary events rate (1000 patients·year: 95%CI)	107.1 (81.2–141.1)	75.4 (55.6–102.2)
Number of deaths	17	20
Deaths rate (1000 patients·year: 95%CI)	36.4 (22.7–58.3)	36.8 (23.8–56.8)

C-HD, conventional haemodialysis; E-HD, electrolyzed water haemodialysis.

PAD, peripheral artery disease (with surgical procedure).

**Table 4:** Cox proportional hazards model analysis for the composite primary endpoints.

	Univariate HR	95%CI		P value	Multivariate HR	95%CI	P value
E-HD	0.687	0.454-1.039		0.076	0.593	0.384–0.916	0.019
HD vintage	1.000	0.997–1.002		0.824			
Age	1.036	1.017–1.055		0.000	1.014	0.993–1.036	0.183
Gender (female)	0.698	0.454–1.074		0.102			
History of CVD	3.085	2.040–4.665		0.000	3.037	1.977–4.665	0.000
non DM	0.865	0.569–1.314		0.497			
BMI	0.987	0.933–1.044		0.644			
Pre SBP	0.999	0.990–1.007		0.783			
Albumin	0.195	0.101–0.377		0.000	0.328	0.160–0.674	0.002
CRP	1.266	1.017–1.576		0.035	1.323	1.005–1.740	0.046
Hg	0.893	0.741–1.075		0.230			

E-HD, electrolyzed water haemodialysis; HD, haemodialysis; CVD, cardio-cerebral vascular disease; DM, diabetes mellituss; BMI, body mass index; Pre SBP, pre-dialysis systolic blood pressure; CRP, C-reactive protein; Hg, hemoglobin.

## Discussion

This prospective observational study primarily aimed to examine the clinical effects of the addition of H_2_ to HD dialysate (an average of 30–80 ppb of H_2)_, which was delivered continuously through the dialyzer membrane to the blood during treatment, as reported elsewhere^10^. During the mean observation period of 3.28 years, the study results revealed E-HD as an independent significant factor for reducing the risk of the primary events of all-cause mortality and development of non-lethal cardio-cerebrovascular events. In the study, all HD systems employed an endotoxin-eliminating filter system. Thus, the different clinical profiles between the two groups, patients on E-HD and those on C-HD, reflects the influence of H_2_ during HD.

The mechanisms by which E-HD delivers clinical benefits remain to be elucidated, since there were no differences in dialysis-related clinically relevant parameters between the two groups during the study. However, we could speculate several possibilities. The observation that the amelioration of post-dialysis hypertension (SBP ≥ 140 mmHg) in E-HD patients may suggest an idea to elucidate the benefits of E-HD, because intra-dialysis systolic hypertension, as well as high SBP, are well-known risk factors for all-cause mortality in HD patients^16,17^. On the other hand, low SBP (<110 mmHg) has also been reported as a risk for excessive mortality^18^. Interestingly enough, there were no differences during the course of the study in post-dialysis SBP levels among the patients with SBP < 140 mmHg at baseline (Fig. 2). Furthermore, there were no differences between the two groups in the proportion of patients with SBP < 110 mmHg (Supplementary Fig. S1). Thus, taken together the observations, the improved post-dialysis BP may have played a role, at least partially, for the better outcomes in patients with post-dialysis hypertension

Other possible mechanisms could be suggested in the previous studies, i.e., increased reduced albumin redox status by acute as well as long-term E-HD^11,19^, improved patients’ anti-oxidative capacity, amelioration of micro inflammation with reduction of pro-inflammatory cytokines^12,13^, and suppression of T-cell damage^14^. These possible mechanisms need to be clarified in the context of patients’ clinical outcomes in the future.

The mitigating effect on elevated SBP, as observed in the present study and previous studies, is very unique. We speculate that the primary mechanism of BP reduction could not be attributed to changes in fluid volume, since there were no significant differences in body weight after HD. Rather, the primary mechanism of BP reduction might be related to vasodilation or to a reduction in vascular resistance. Recent studies in deoxycortisterone acetate (DOCA)-salt hypertension have revealed a crucial role of superoxide anion release from macrophages in mesenteric peri-arteries, due in part to impaired function of the Alpha 2-adrenergic autoreceptors^20^, which provide negative feedback on the release of norepinephrine from the sympathetic nerves associated with the mesenteric arteries. The mesenteric arteries constitute a major resistance arterial bed for BP regulation. In addition, one fourth of the systemic blood volume is present in the splanchnic circulation. Therefore, an increase in arteriolar resistance will elevate the arterial BP, and an increase in the mesenteric venomotor tone will lead to an increase in the cardiac venous return and the cardiac load due to a decrease in the venous capacity^20,21^. The combination of these two pathological processes results in a severe cardiac load. Interestingly, a recent study showed that the chemokine (C-C motif) receptor type 2 blockade suppresses vascular macrophage infiltration and reduces blood pressure^22^. Upon the observation that MCP-1 decreased in E-HD patients in the previous study, it is possible to speculate the possible action of E-HD on macrophage of patients^10^. The question of whether the HD procedure activates the residential macrophages, or activates extrinsic macrophages to infiltrate the mesenteric vascular area, needs to be addressed.

There are several issues and limitations in this study. First, the observed results in the E-HD group were slightly complicated, i.e., the rate of the primary composite endpoint was lower in the E-HD group than in the C-HD group, although the rate of death was not different between the groups. In univariate analysis of the Cox proportional hazards model, E-HD was not a strong factor for the primary endpoint, although multivariate analysis showed E-HD to be a strong factor after adjusting for confounding factors. Regarding the reasons for this, we speculate that a potential bias existed in the patients who were allocated to the E-HD group in that these patients had a relatively higher incidence of CVD history. This may have influenced the results of the univariate-analysis, since the presence of a CVD history was the most influential risk factor for the occurrence of the primary endpoint. To clarify this point, we performed a sub-analysis on this profile according to the presence or absence of CVD history. And it was revealed that E-HD was a significant factor for reducing the risk of primary endpoint in patients without history of CVD (HR: 0.455; p = 0.010) by univariate as well as multivariate analysis (Supplementary Tables S1 and 2), which indicates the clinically significant impact of E-HD.

Second issue is the levels of H_2_ of HD solution. The H_2_ levels of the present dialysates were in the range of 30–80 ppb, and no adverse effects were observed with respect to an H_2_ load within this range. Upon the report that there are generation of H_2_ in average of 24 ml/min in healthy human (approximately over 15 mmol daily) in the colon, and that they are absorbed into body^23^, the given H_2_ during the single session of HD, which we estimated approximately as much as 2.5 mmol, seemed to be within the physiological range. Therefore, it remains unknown whether the applied H_2_ levels were best in regards to provide clinical effects, and higher levels of H_2_ may offer additional clinical benefits without any adverse effects needs to be investigated.

Third, we could not conclude the influence of E-HD on clinical symptoms in this study. Of note is that during the clinical course, post-dialysis hypertension was ameliorated with significant reductions of anti-hypertensive agents in the patients on E-HD. However, patient selection in the present study was conducted according to the attending physician’s preference; therefore, the observed phenomena such as decrease in BP and improved subjective symptoms of fatigue and pruritus during the course, have remained speculative.

And lastly, there was a statistical difference in the age between the two groups in the present study, e.g. the E-HD group was 3.4 years younger than C-HD. Although we employed the age for multivariate analysis of Cox proportional hazards model analysis, this might have influenced the event rate in the real world. A randomized clinical study is critically needed to address these issues in the future.

H_2_ as biological gas has potentials in clinical medicine. However H_2_ volatile gas, is not easy to handle in the clinical setting. The technique of water electrolysis has made it possible to apply H_2_ very safe to generate H_2_ dissolved water for real HD therapy. We think that this innovative treatment could open a new therapeutic possibility beyond the conventional HD.

## Method

### Study design and participants

A non-randomized, non-blinded, prospective observational study was conducted to evaluate the clinical impact of the E-HD system (UMIN-ICDR Clinical Trial: Study Title: “Prospective observational study of the clinical effect of haemodialysis using electrolyzed water”; Unique ID issued by UMIN: UMIN000004857, Date of disclosure of the study information: 2011/01/11, Link to view the page (ICDR): https://upload.umin.ac.jp/cgi-bin/icdr_e/ctr_view.cgi?recptno = R000005491).

The primary composite endpoints included all-cause mortality, and concomitant disease such as cardiac disease (heart failure or myocardial infarction requiring hospitalization, coronary artery disease requiring invasive therapy), stroke (symptomatic cerebral hemorrhage or cerebral infarction confirmed by diagnostic imaging), and obstructive arteriosclerosis requiring leg amputation.

The study used a non-randomized design, and the candidate patients were selected by decision of the patient’s physician. In two centers (KH and NMH), candidates for the E-HD group were selected by chief physicians; subsequently, matched control patients in the C-HD group were selected from the rest of the patients in the respective centers in terms of demographic background such as age and sex. In two of the study centers (HMC and HHC), all patients were selected for the E-HD group since the centers were to employ a central E-HD system to completely replace the conventional HD system. In three study centers in which the E-HD system was not available (NH, TJC, GJC), more than one patient was selected as part of the matched control group to the E-HD group of the above four centers in terms of age and sex as much as possible. Patients who were receiving on-line hemodiafiltration or combination therapy with peritoneal dialysis, and potential subjects with serious disease at the time of enrollment, i.e., severe heart failure (New York Heart Association III/IV), severe liver disease, psychological problems, dementia, malignant disease within the previous 3 months, or an evidently poor systemic condition with an evidently poor short-term prognosis, were excluded from this study. History of CVD included cardiac disease, stroke (these definitions were comparable to those of the primary composite endpoints mentioned above), and symptomatic peripheral arterial disease requiring medical intervention.

The study was approved by the Ethics Committee of Fukushima Medical University (No. 1155: Supplementary file of study protocol), and the clinical investigation was conducted according to the principles expressed in the Declaration of Helsinki. Written informed consent was obtained from all patients registered.

### Data collection

All patients were monitored for subjective symptoms and objective signs during the study period. Blood pressure was measured using a sphygmomanometer on the upper arm with the patient in a supine position just before starting each HD session, and data were recorded into the clinical record. Iron, erythropoiesis-stimulating agents (ESA) to correct anemia, and agents to control calcium and phosphate were administered according to the guidelines of the Japanese Society of Dialysis Treatment^24,25^. Antihypertensive agents and adjustment of body weight after HD (dry weight) were administered as needed by the attending physician. Quantities of antihypertensive agents were standardized using DDD^15^. Regular monitoring of blood was performed at the first HD session of the week (Monday or Tuesday) at least once a month to monitor dialysis status. Patients were requested to fill out a self-assessment questionnaire every 6 months, which asked about the subjective symptoms of fatigue on the HD day and pruritus according to the following criteria: Fatigue (subjective level and daily activities)–Grade 1: Intense fatigue/Disturbed activity and required rest; Grade 2: Moderate fatigue/Reduced activity; Grade 3: Mild fatigue/Normal activity; Grade 4: Tireless/Normal activity; Grade 5: Inexhaustible/Active; Pruritus (subjective intensity and frequency)–Grade 1: Intense/Always; Grade 2: Moderate/Sometimes; Grade 3: Mild/Rarely; Grade 4: None/None. Levels of H_2_ were determined using the gas chromatograph with a semiconductor detector (TRIlizer mBA-3000, Taiyo Instruments Co., Osaka, Japan) according to the manufacturer’s instruction, as reported elsewhere^11^.

All data generated or analysed during this study are included in this published article.

### Statistical Methods

The target sample size of the original study (n = 70 < each) was based on an estimated event-free rate of 10% differences at 3 years between groups with 1:1 ratio between them, and calculated from the rationale that a statistical power of 90% and the alpha level 0.05, using a two-sided log-rank test.

All values are expressed as the mean ± standard deviation (SD) or median (interquartile range) as appropriate. For comparisons between the two groups, Student’s t-test or the Mann-Whitney *U* test was used for continuous variables and chi-square test or Fisher’s exact test was used for nominal variable, as appropriate. Values of p < 0.05 were considered statistically significant. Data were statistically analyzed using IBM SPSS Statistics version 22.0 for Windows (Chicago, IL, USA).

### H_2_ delivery HD system

**Figure 4:**
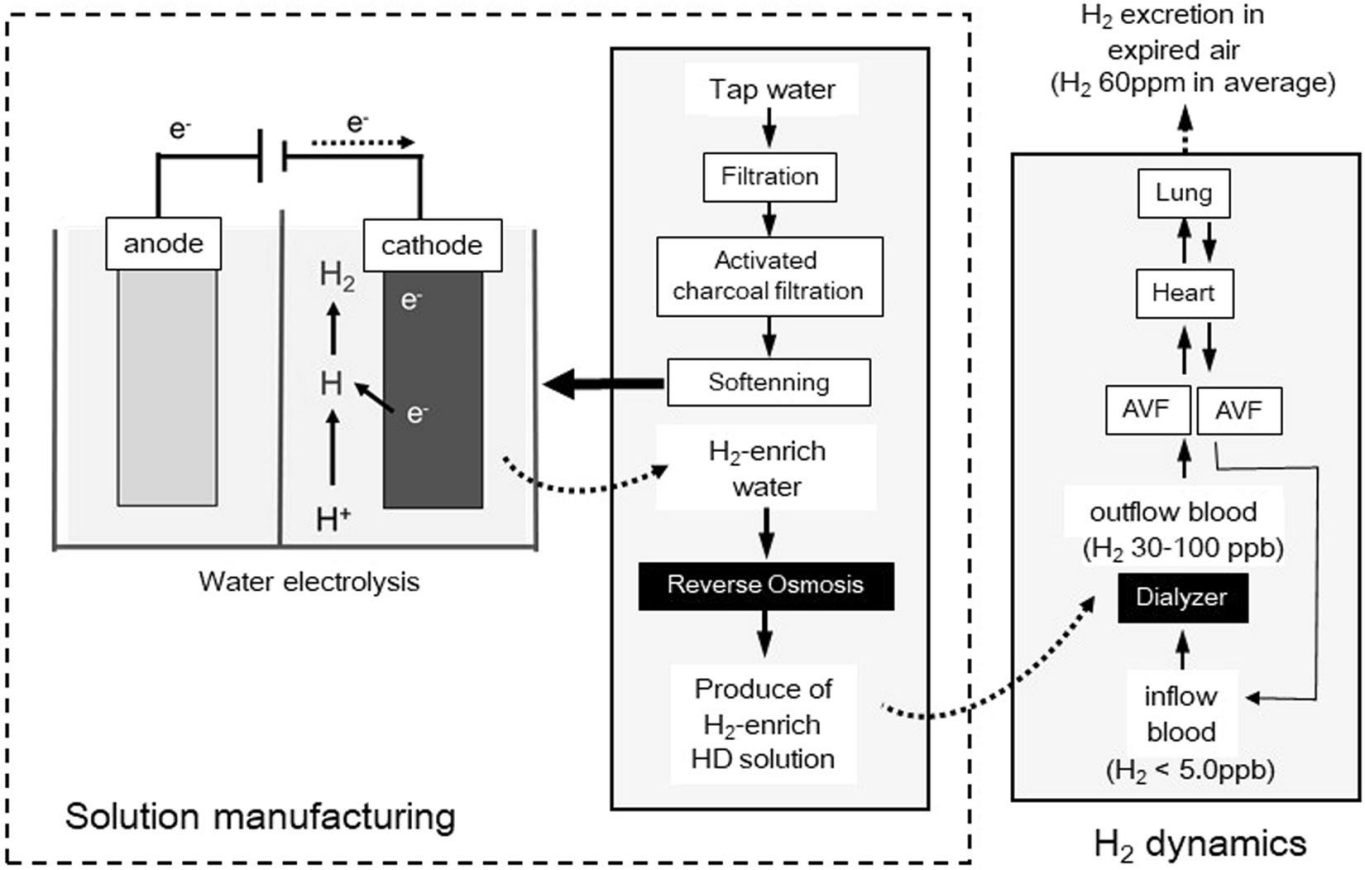
Manufacturing process of haemodialysis solution in the E-HD and H_2_ dynamics during treatment by E-HD. Abbreviations: E-HD, electrolyzed water haemodialysis; e-, electron; AVF, arterio-venous fistula.

Figure 4 details of the system have been reported previously^10,11^. Briefly, test solutions were prepared as follows: pre-filtered water was processed using activated charcoal filtration and water softening to supply the HD-24K water electrolysis system (Nihon Trim, Osaka, Japan), where water was electrolyzed by direct current supply to the anode and cathode electrode plates. Water on the anode side was drained, and water from the cathode side (electrolyzed water) was collected to supply the reverse osmosis equipment (MH500CX; Japan Water System, Tokyo, Japan) at 500 mL/min. The intensity of electrolysis was adjusted to maintain a pH of 10.0. The reverse osmosis water produced by the water electrolysis system was supplied to prepare the HD solution. The composition of the inflow H_2_-HD solution was the same as the standard HD solution with the exception of the presence of dissolved H_2_ in the H_2_-HD, and there were no differences in terms of electrolytes levels and pH, as compared to the standard HD solution, as reported elsewhere^8,11^. Whereas regarding H_2_ levels of control group, dialysate and blood H2 levels were less than 1 ppb^11^.

The present E-HD system could deliver H_2_ (30–80 ppb)-enriched dialysis solution. H_2_ levels of inflow blood and HD solution reached an equivalent state in the dialyzer, and the H_2_ level of outflow blood from dialyzer showed approximately the same as that of inflow H_2_-HD solution under QB 200 ml/min and QD 500 ml/min. Therefore, H_2_ load to patient is determined by time of HD treatment and H_2_ levels of HD solution if QB and QD are fixed, i.e., it is estimated that about 1.2 mmol of H_2_ is loaded in case of 4 hour-treatment, and HD solution with 50 ppb H_2_. Regarding the H_2_ dynamics in the body, previous studies^10,11^ revealed that no changes were found in H_2_ levels of inflow blood after 4-hour treatment, and there were increases of constant H_2_ levels in expired air of patients by treatment, and they soon returned to the basal levels by stop of treatment. Therefore, it is supposed that delivered H_2_ into blood during the HD treatment is mostly excreted from lung during the time on HD.

## Electronic supplementary material

Supplementary Table S1., S2., Fig. S1.
