# Supplementary material for: Novel haemodialysis (HD) treatment employing molecular hydrogen (H2)-enriched dialysis solution improves prognosis of chronic dialysis patients: A prospective observational study
Source: Sci Rep. 2018 Jan 10;8:254. doi: 10.1038/s41598-017-18537-x (PMC5762770; doi:10.1038/s41598-017-18537-x)
Supplement: Supplementary file 1 — Supplementary Table S1., S2., Fig. S1. [file 41598_2017_18537_MOESM1_ESM.pdf]

Title:

Novel haemodialysis (HD) treatment employing molecular hydrogen (H_2)-enriched dialysis solution improves prognosis of chronic dialysis patients: A prospective observational study

Authors:

Masaaki Nakayama, Noritomo Itami, Hodaka Suzuki, Hiromi Hamada, Ryo Yamamoto, Kazumasa Tsunoda, Naoyuki Osaka, Hirofumi Nakano, Yukio Maruyama, Shigeru Kabayama, Ryoichi Nakazawa, Mariko Miyazaki, Sadayoshi Ito.

Supplementary Table S1. Occurrence of composite primary endpoints by absence or presence of CVD history

	CVD (-), n=220	CVD (+), n=89
Patients on C/E-HD	112/108	36/53
Occurrence of composite primary endpoints (%), and proportion of C/E-HD	48 (21.8%)<br>32/16	43 (48.3%)<br>18/25

CVD, cardio-cerebral-vascular disease;
C/E-HD, control / electrolozed water haemodialysis

Supplementary Table S2. Cox proportional hazards model analysis for the composite primary endpoints in cases without CVD history (a) and those with CVD history (b)

(a)

	Univariate HR	95%CI	P value		Multivariate HR	95%CI	P value
E-HD	0.455	0.249-0.831	0.010		0.480	0.257-0.896	0.021
Age	1.044	1.020-1.068	0.000		1.020	0.993-1.048	0.153
Albumin	0.134	0.047-0.374	0.000		0.186	0.055-0.631	0.007
CRP	1.186	0.873-1.611	0.276		1.203	0.825-1.753	0.337

(b)

	Univariate HR	95%CI	P value		Multivariate HR	95%CI	P value
E-HD	0.388	0.417-1.405	0.388		0.699	0.374-1.307	0.699
Age	1.001	0.972-1.041	0.739		0.990	0.955-1.027	0.605
Albumin	0.439	0.190-1.016	0.054		0.625	0.231-1.690	0.355
CRP	2.155	1.316-3.528	0.002		1.960	1.113-3.453	0.020

CVD, cardio-cerebral-vascular disease;
E-HD, electrolozed-water haemodialysis

Supplementary Fig. S1. Changes in post-dialysis systolic blood pressure (SBP) in patients with SBP more than 140 mmHg at baseline (a), and those with less than 140 mmHg at baseline (b) .

(a)

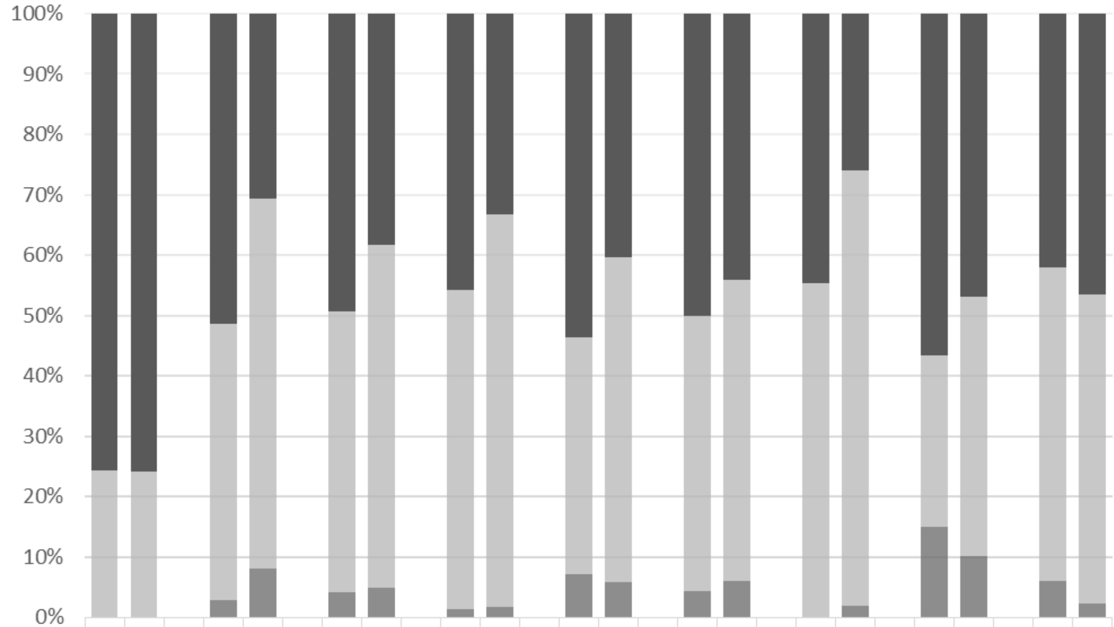

	C0	E0	C6	E6	C12	E12	C18	E18	C24	E24	C30	E30	C36	E36	C42	E42	C48	E48
150<	59	47	37	19	35	23	33	19	37	21	34	22	29	13	30	23	21	20
110-150	19	15	33	38	33	34	38	37	27	28	31	25	36	36	15	21	26	22
<110	0	0	2	5	3	3	1	1	5	3	3	3	0	1	8	5	3	1

(b)

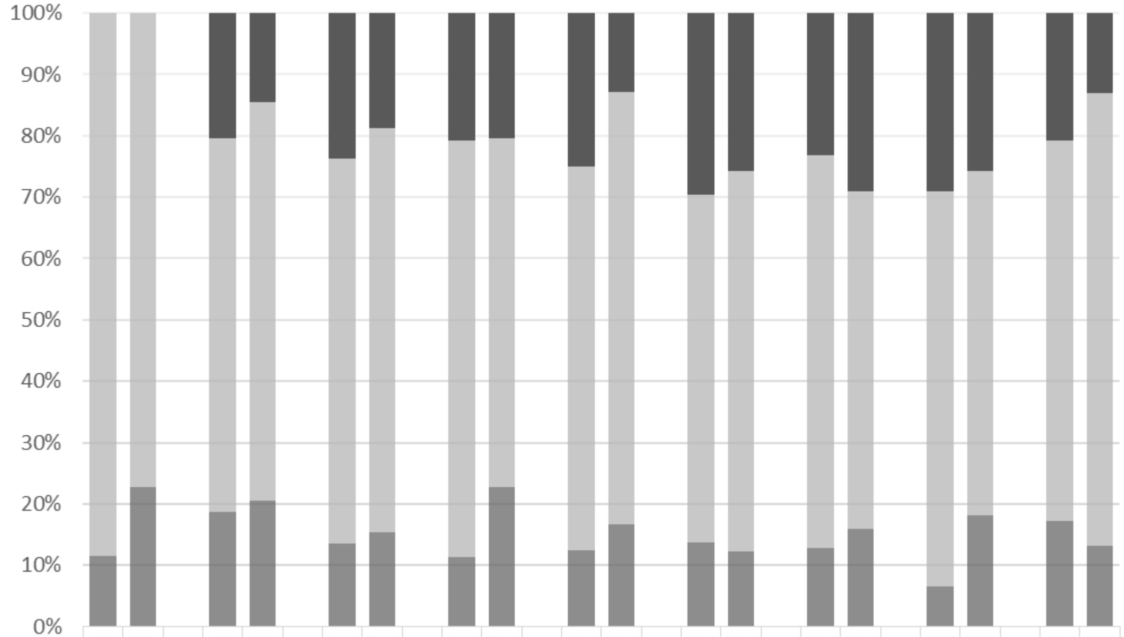

	C0	E0	C6	E6	C12	E12	C18	E18	C24	E24	C30	E30	C36	E36	C42	E42	C48	E48
150<	0	0	13	14	14	17	11	18	12	10	13	19	9	20	9	17	6	8
110-150	61	75	39	63	37	60	36	50	30	55	25	46	25	38	20	37	18	45
<110	8	22	12	20	8	14	6	20	6	13	6	9	5	11	2	12	5	8

(mmHg) (number of patients of respective categories)

No statistical differences were found in the proportion of hypotensive patients with post-dialysis SBP less than 110 mmHg during the study in the both groups (a, b).
Abbreviations: C, E: conventional, electrolyzed water haemodialysis group, respectively
(0) denotes month of the study.
